# Supplementary material for: Two-tiered enforcement of high-fidelity DNA ligation
Source: Nat Commun. 2019 Nov 28;10:5431. doi: 10.1038/s41467-019-13478-7 (PMC6882888; doi:10.1038/s41467-019-13478-7)
Supplement: Supplementary file 1 — Supplementary Information [file 41467_2019_13478_MOESM1_ESM.pdf]

Supplementary Information

Two-Tiered Enforcement of High-Fidelity DNA Ligation

Percy Tumbale, Thomas J. Jurkiw, Matthew J. Schellenberg, Amanda A. Riccio, Patrick J. O'Brien, and R. Scott Williams

Correspondence: williamsrs@niehs.nih.gov or pjobrien@umich.edu

PDF Contents:

Supplementary Figure 1. Initial characterization of the E346A/E592A LIG1 mutant

Supplementary Figure 2. Steady-state substrate dependence for WT and E346A/E592A LIG1

Supplementary Figure 3. Steady-state and pre-steady-state magnesium dependencies for ligation

Supplementary Figure 4. Steady-state DNA dependences under conditions of 1 mM Mg^{2+}

Supplementary Figure 5. X-ray structures of $LIG1^{E346A/E592A}$ and structural comparison to the WT

Supplementary Figure 6. Effect of APTX on steady-state ligation by LIG1

Supplementary Figure 7. Catalytic commitment of LIG1 under single-turnover conditions

Supplementary Figure 8. Purification and characterization of WT and H260N APTX

Supplementary Figure 9. Model for single turnover ligation in the presence of H260N APTX

Supplementary Figure 10. Biochemical characterization of $LIG1^{E592R}$

Supplementary Table 1. Oligonucleotides for crystallization and ligation assays

Supplementary Table 2. Steady-state kinetic parameters for ligation at 20 mM Mg^{2+}

Supplementary Table 3. Kinetic parameters for varying the concentration of Mg^{2+}

Supplementary Table 4. Kinetic parameters for ligation of DNA substrates at 1 mM free Mg^{2+}

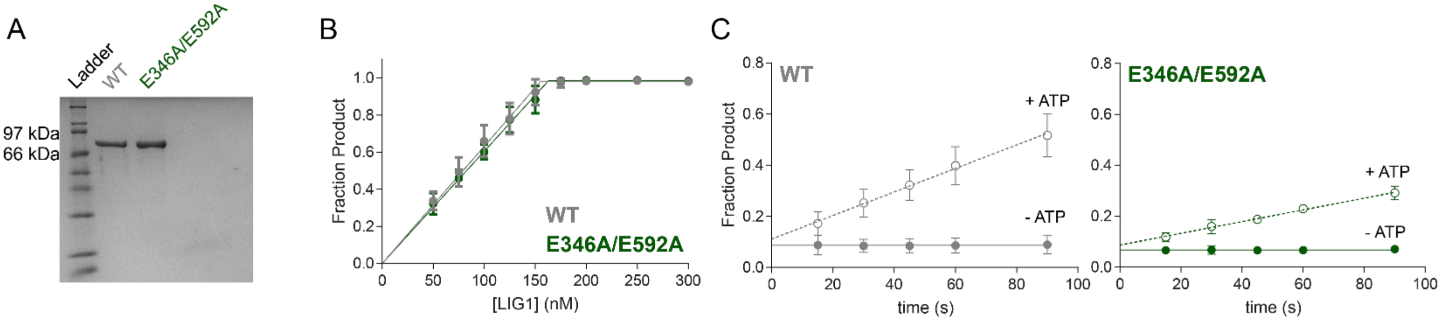

Supplementary Figure 1. Initial characterization of the E346A/E592A LIG1 mutant. (A) Purity of recombinant LIG1^{WT} and LIG1$^{E346A/E592A}$ was assessed by separating ~1 µg of the LIG1 proteins and a protein ladder on a 15% polyacrylamide/SDS gel. **(B)** The concentration of active, adenylylated Δ232 LIG1 was confirmed by quantifying the amount of ligated DNA in the absence of added ATP. The nicked DNA substrate was fixed at 150 nM and the nominal concentration of LIG1 protein was varied from 0-300 nM. These data gave active concentrations that deviated ~4% (LIG1^{WT}) and ~8% (LIG1$^{E346A/E592A}$) from the estimated concentrations determined via UV-absorbance. **(C)** Pre-steady-state burst experiments were performed to assess the adenylylation state of the recombinant enzymes. Mixes containing 50 nM LIG1 and either 0 (closed symbols) or 1 µM (open symbols) ATP were allowed to incubate at 37°C for 5 minutes before reactions were initiated with the addition of 500 nM nicked DNA. Differences in the burst amplitude between the ATP-containing and no ATP reactions indicate the presence of deadenylylated enzyme. The ~20% deadenylylated enzyme population was taken into account for calculating k_{cat} in steady-state reactions. All data are reported as the mean ± S.D. of N ≥ 3 replicates. Source data for panels are provided as a Source Data file.

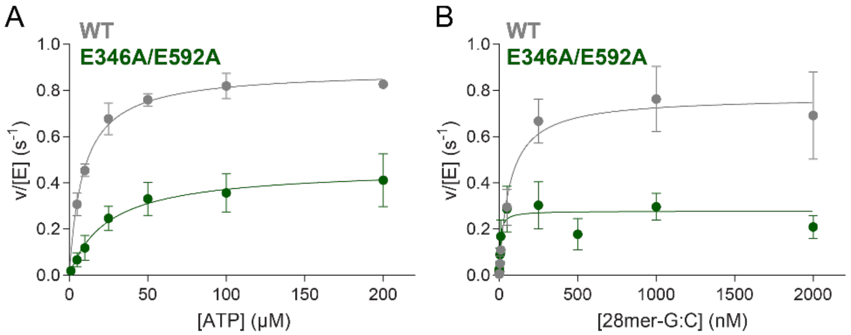

Supplementary Figure 2. Steady-state substrate dependences for LIG1^{WT} and LIG1$^{E346A/E592A}$. The steady-state ATP **(A)** and 28mer-C:G DNA dependences **(B)** were determined for LIG1^{WT} and LIG1$^{E346A/E592A}$. Both dependences were performed with 20 mM $MgCl_2$ and 0.1-10 nM LIG1. 500 nM 28mer-C:G was used for the ATP dependence and 200 µM ATP was used for the DNA substrate dependence. The values obtained from fitting these dependences with the Michaelis-Menten equation are shown in Table S2. All data are reported as the mean ± S.D. of N ≥ 3 replicates. Source data for panels are provided as a Source Data file.

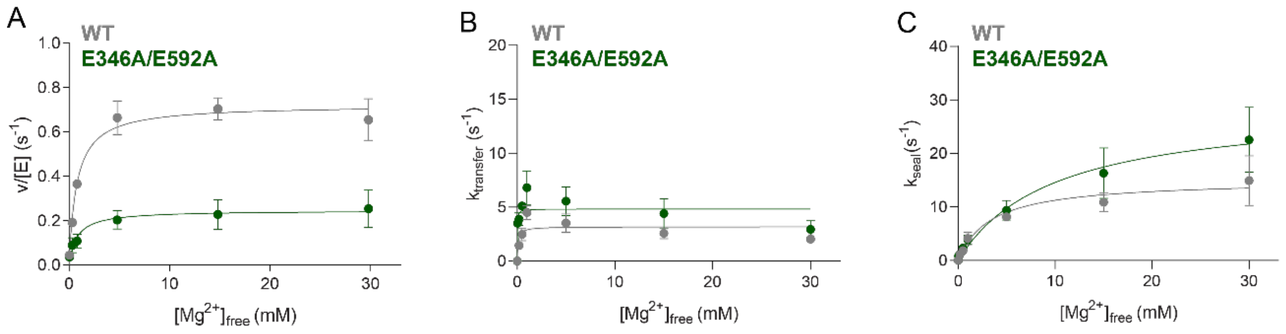

Supplementary Figure 3. Steady-state and pre-steady-state magnesium dependences for ligation. (A) The steady-state Mg^{2+} dependence was determined for $LIG1^{WT}$ and $LIG1^{E346A/E592A}$ with steady-state ligation assays containing 200 µM ATP, 500 nM 28mer-C:G DNA, and 1-5 nM LIG1. The values obtained from fits of the data to the Michaelis-Menten equation are shown in Table S3. Pre-steady-state magnesium dependences were carried out to determine the dependence of adenylyl transfer **(B)** and nick sealing **(C)** on the concentration of magnesium. Reactions containing 800 nM LIG1 and 80 nM 28mer-C:G were performed in the presence of increasing magnesium concentrations using a Kintek Rapid Quench Flow apparatus. Berkeley-Madonna was used to determine the individual rates of adenylyl transfer and nick sealing, which were fit to hyperbolic curves to determine the maximal rates and the K_{Mg} values (Table S3). All data are reported as the mean ± S.D. of N ≥ 3 replicates. Source data for panels are provided as a Source Data file.

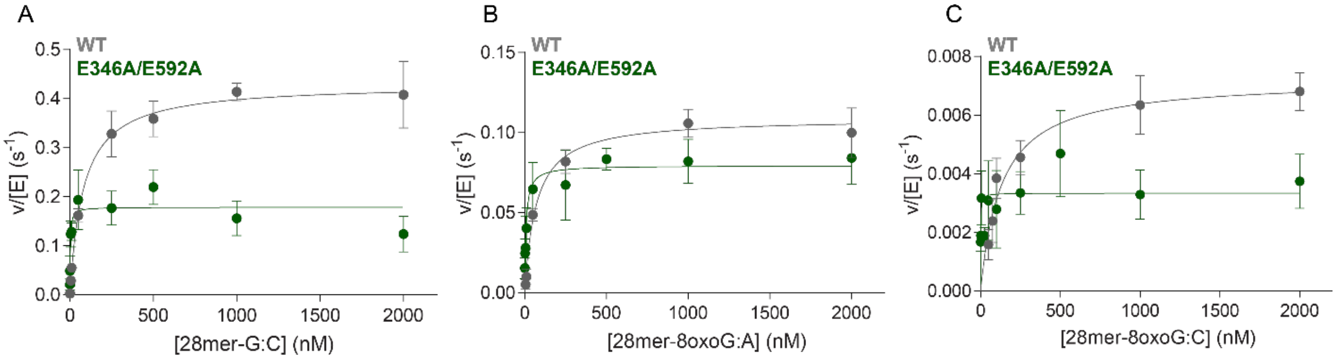

Supplementary Figure 4. Steady-state DNA dependences under conditions of 1 mM free Mg^{2+}. Steady-state substrate dependences were performed for the 28mer C:G **(A)**, 8oxoG:A **(B)**, and 8oxoG:C (**C**) substrates in the presence of 1 mM ATP and 2 mM $MgCl_2$ (1 mM $Mg^{2+}{}_{free}$). Reactions contained 0.1-10 nM of either $LIG1^{WT}$ and $LIG1^{E346A/E592A}$. The data were fit by the Michaelis-Menten equation, which was used to determine the steady-state constants shown in Table S4. All data are reported as the mean ± S.D. of N ≥ 3 replicates. Source data for panels are provided as a Source Data file.

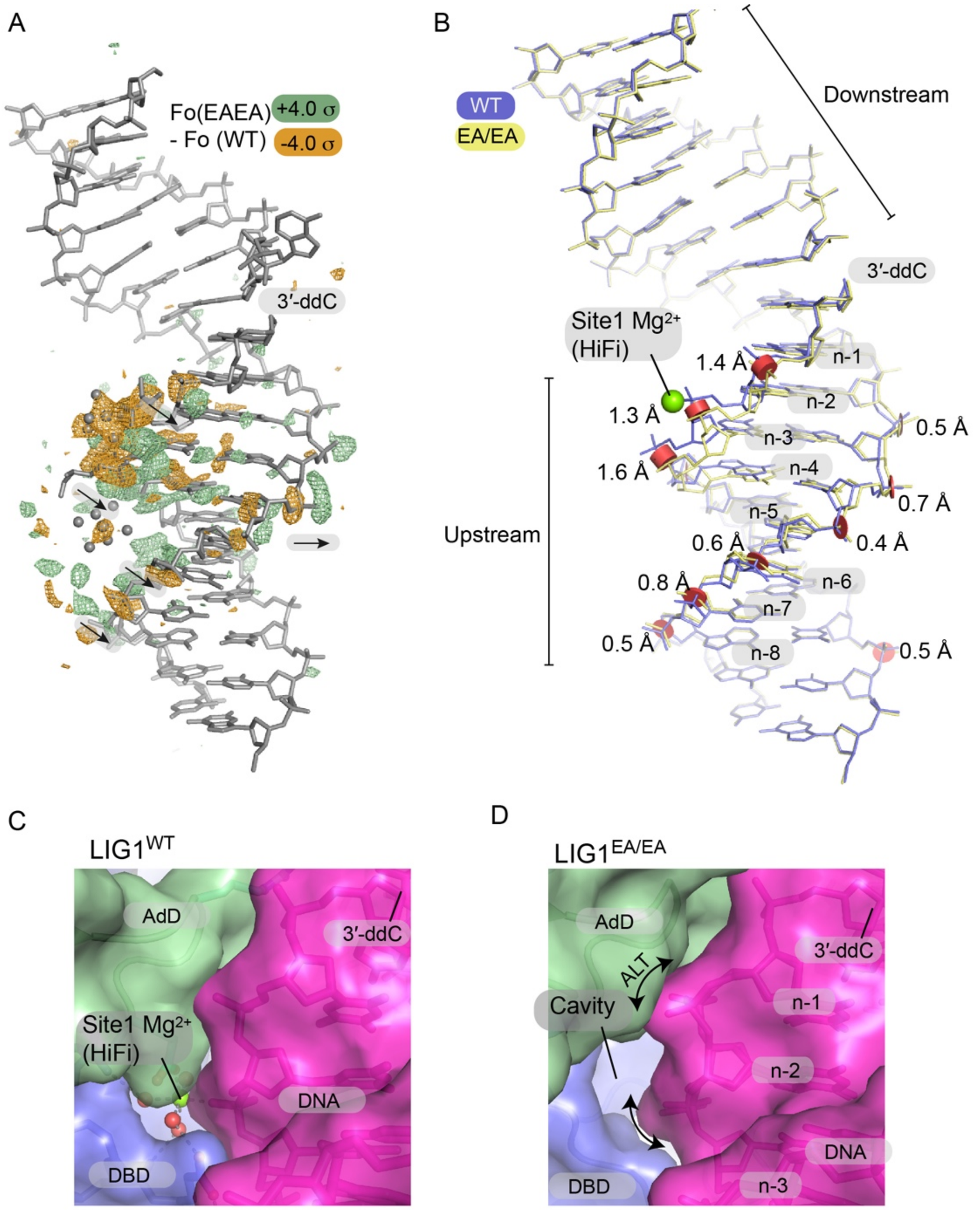

Supplementary Figure 5. X-ray structures of LIG1$^{E346A/E592A}$ and structural comparison to LIG1^{WT}. (A) Model phased $F_{o,mutant}$ (green) - $F_{o,WT}$ (orange) is displayed contoured at 4.0 σ for the crystallographic asymmetric unit. Arrows indicated concerted repositioning of the upstream strand in the LIG1$^{E346A/E592A}$•DNA complex. **(B)** Structural overlay of the WT and mutant DNA complexes. Scaled red cylinders show relative displacements of the DNA strand and reflect RMSD of the structural overlay performed in Pymol. **(C)** Surface representations of the intersection of the AdD, DBD and DNA at the intersection marked by the HiFi site. The Mg^{HiFi} ion is a linchpin at the nexus of the DNA-DBD-AdD junction. The molecular interactions enforce the conformation of bound DNA substrate by complementarity of protein domains and DNA backbone. **(D)** Surface representation of the LIG1$^{E346A/E592A}$ mutant ligase active site. A cavity created by removal of Mg^{HiFi} ligands accommodates flexible 3′ strand binding.

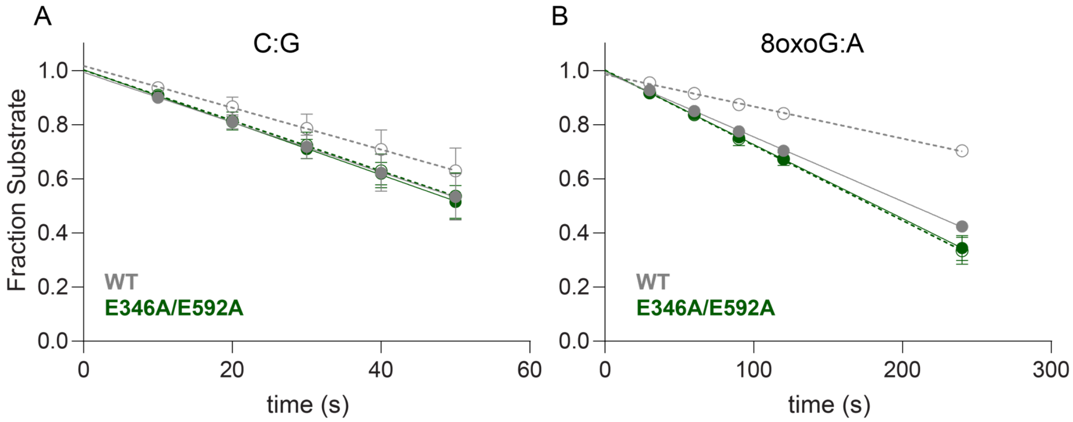

Supplementary Figure 6. Effect of APTX on steady-state ligation. Steady-state ligation reactions were performed in the presence of 1 mM ATP and 1 mM Mg^{2+}_{free} and contained 10 nM LIG1 and 500 nM of either the 28mer-C:G **(A)** or 28mer-8oxoG:A **(B)** DNA substrate. APTX was either absent (closed symbol, solid line) or added at a concentration of 1 nM (open symbol, dashed line) to the reactions. Substrate disappearance is plotted as a function of time for reactions containing either WT or $LIG1^{E346A/E592A}$ mutant LIG1 (mean ± S.D.; N ≥ 3). The amount of ligation suppressed by APTX activity was calculated as the percent change in the initial ligation rate upon the addition of APTX (Figure 4B). Source data for panels are provided as a Source Data file.

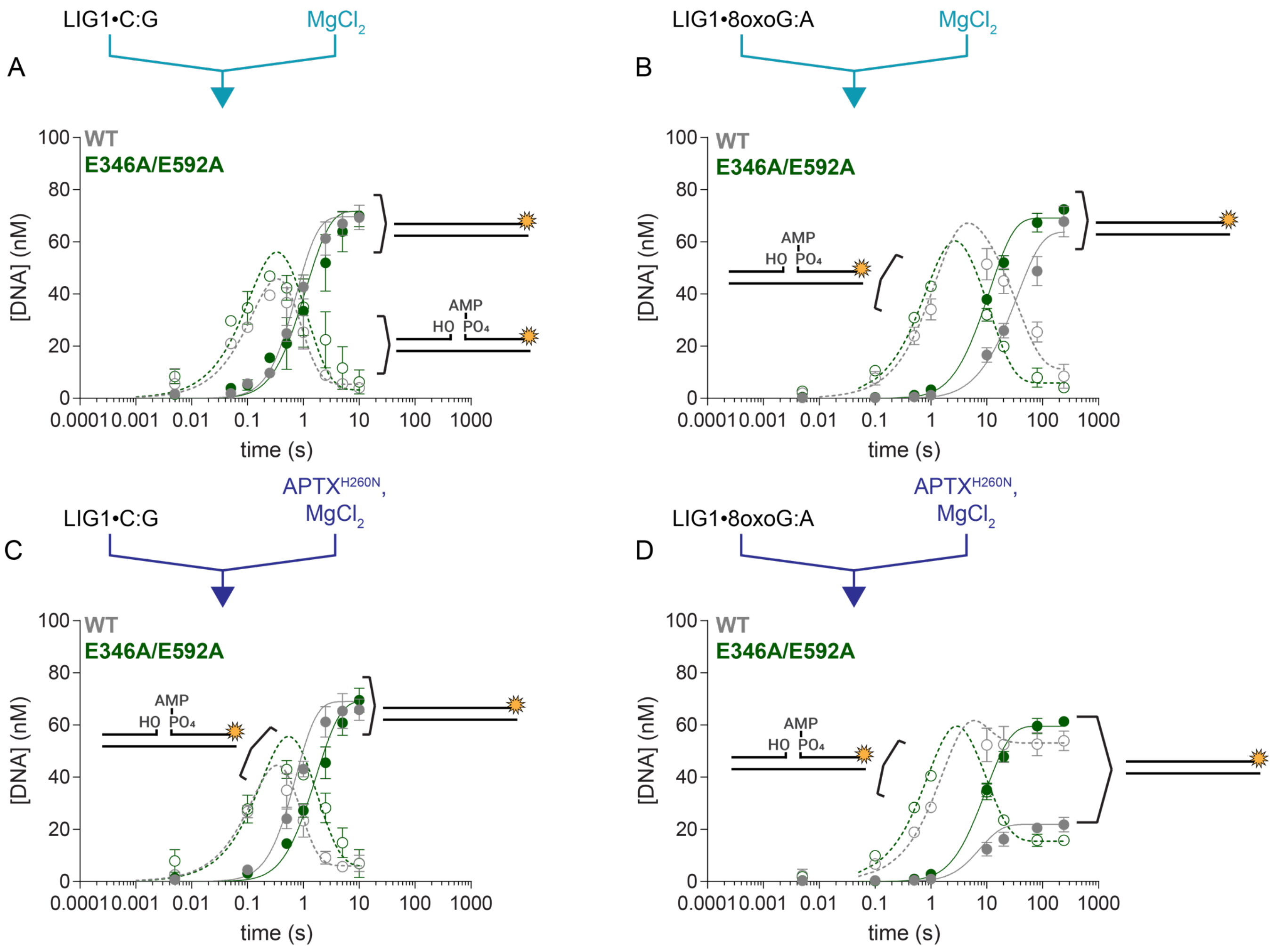

Supplementary Figure 7. Catalytic commitment of LIG1 under single turnover conditions. Single-turnover reactions were carried out by mixing a solution containing either 1600 nM $LIG1^{WT}$ or $LIG1^{E346A/E592A}$, 160nM 28mer-G:C **(A,C)** or 28mer-8oxoG:A **(B,D)** and with a separate solution containing 4 mM $MgCl_2$. Both reaction mixes contained 1 mM ATP, leading to a concentration of the free magnesium ion of 1 mM upon mixing. Reactions were carried out in the presence **(C,D)** or absence **(A,B)** of 800nM $APTX^{H260N}$ in the $MgCl_2$ solution. Product formation is represented by closed symbols, while intermediate formation is denoted by open symbols (mean ± S.D.; N ≥ 3). Curve fits shown for both product (solid line) and intermediate (dashed lines) formation were obtained by fitting the data using Berkeley-Madonna with a two-step irreversible model with an added dissociation step after adenylyl-transfer (Figure S9). The catalytic commitment for both $LIG1^{WT}$ and $LIG1^{E346A/E592A}$ was calculated for reactions with $APTX^{H260N}$ (Figure 4F). Source data for panels are provided as a Source Data file.

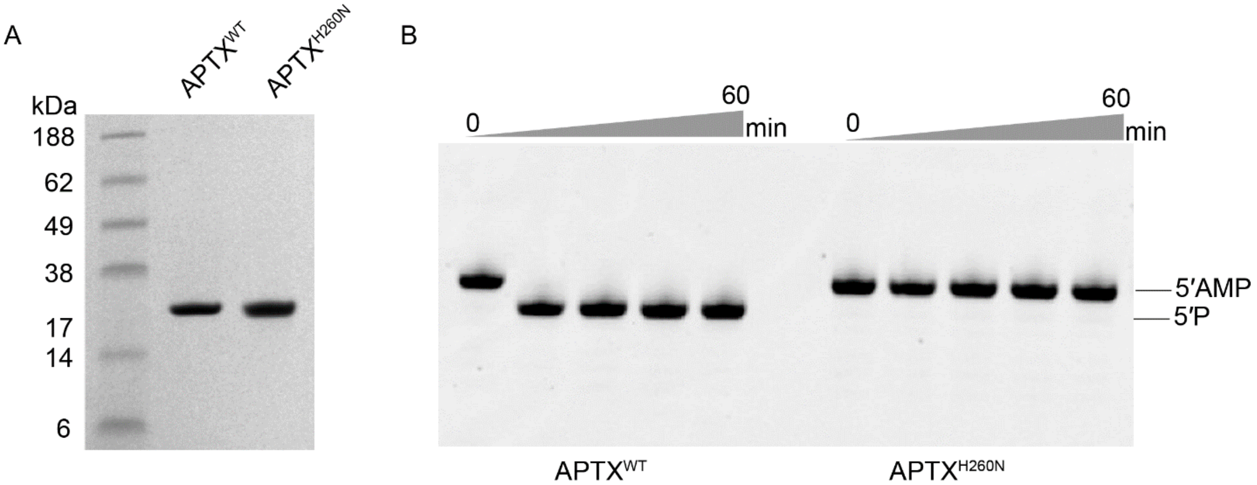

Supplementary Figure 8. Purification and characterization of WT and H260N APTX. (A) Purified recombinant APTX^{WT} (residues 165-342) or APTX^{H260N} catalytic domain (residues 165-342). **(B)** APTX deadenylylation reactions were performed by incubating 10 nM nicked adenylated substrate (Tumbale et al, 2018, ref 28) with 100 nM APTX^{WT} or APTX^{H260N}. The H260N substitution inactivates the enzyme. Source data for panels are provided as a Source Data file.

Supplementary Figure 9. Model for single turnover ligation in the presence of H260N APTX. The script for data fitting with Berkeley Madonna is provided and a schematic of the individual steps is provided as an inset.

```
METHOD RK4

STARTTIME = 0
STOPTIME=100
DT = 0.02

d/dt(ES) = -ktransfer*ES
d/dt(EI) = ktransfer*ES - EI*(kseal + koff)
d/dt(EI2) = koff*EI
d/dt(EP) =  kseal*EI

init ES = 78
init EI = 0
init EI2 = 0
init EP = 0

ktransfer = 0.01
kseal = 0.008
koff = 0.008

Product = EP
Intermediate = EI + EI2
```

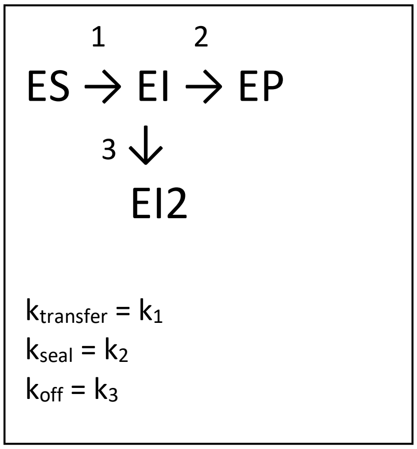

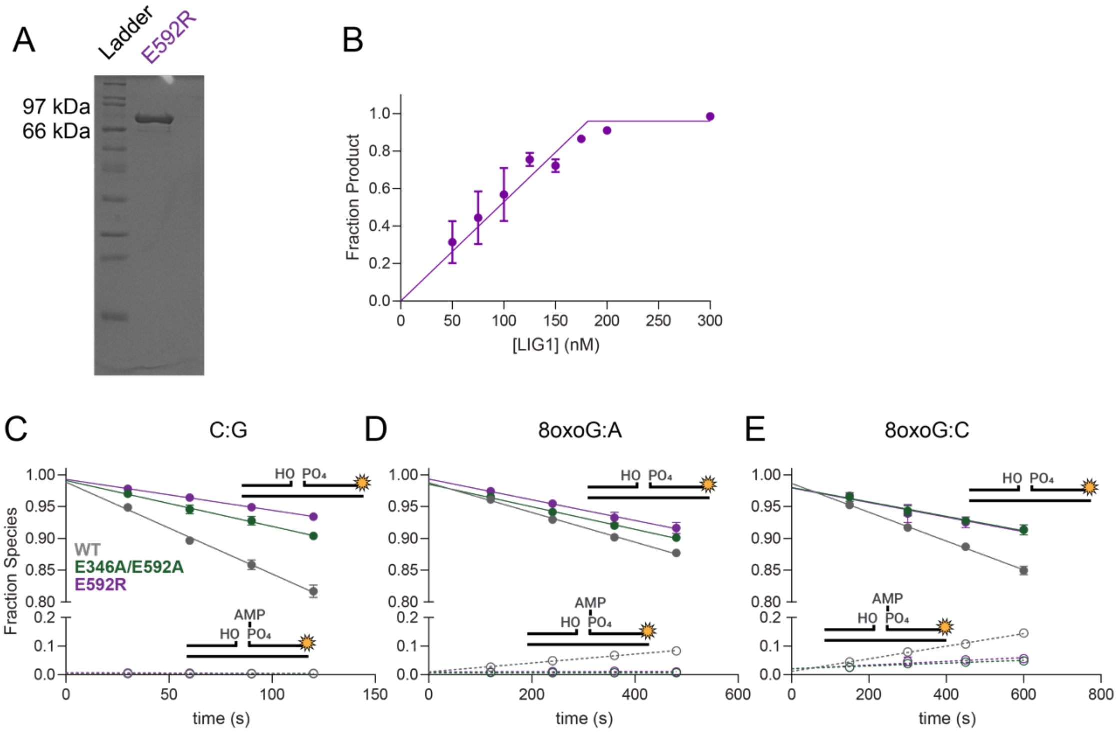

Supplementary Figure 10 Biochemical characterization of $LIG1^{E592R}$. (A) Purity of recombinant $LIG1^{E592R}$ was assessed by separating ~1 µg of the protein and a protein ladder on a 15% polyacrylamide/SDS gel. **(B)** The concentration of active, adenylylated Δ232 $LIG1^{E592R}$ was confirmed by active site titration as in Figure S1B, giving active concentrations that deviated ~15% from the estimated concentrations determined via UV-absorbance. **(C-E)** Steady-state ligation assays were performed with WT and mutant LIG1 to determine the fraction of abortive ligation (Figure 6A). Reactions were performed with 10 or 50 nM LIG1 and 2 µM of the C:G (**C**), 8oxoG:A (**D**) or 8oxoG:C (**E**) substrate in the presence of 1 mM ATP and 2 mM $MgCl_2$ (1 mM Mg^{2+}_{free}). All data are the mean ± S.D. of ≥ 3 replicates. Source data for panels are provided as a Source Data file.

Oligo number	Oligo Name	5′ modification	Sequence	3′ modification
1	18merDown		5’ GTCCGACGACGCATCAGC 3’	
2	11mer3'ddCUP		5' GCTGATGCGT(ddC) 3'	ddC
3	7mer5'PDown	phosphate	5' P-GTCGGAC 3'	
4	11mer3'OHUP		5' GCTGATGCGTC 3'	
5	18mer8oxoG:ADown		5' GTCCGACAACGCATCAGC 3'	
6	11mer8oxoGUp		5' GCTGATGCGT(8oxoG) 3'	8oxoG
7	Up13OH		5’ GTGCTGATGCGTC 3’	
8	DownP15-FAM	phosphate	5’ P-GTCGGACTGATTCGG-FAM 3’	6-FAM
9	Temp28		5’ CCGAATCAGTCCGACGACGCATCAGCAC 3’	
10	Up13OH-8oxoG		5’ GTGCTGATGCGT(8oxoG) 3’	8oxoG
11	Temp28-C		5’ CCGAATCAGTCCGACCACGCATCAGCAC 3’	
12	Temp28-A		5’ CCGAATCAGTCCGACAACGCATCAGCAC 3’	

Supplementary Table 1. Oligonucleotides for crystallization and ligation assays

	28mer-C:G	
	WT	E346A/E592A
k_{cat} (s^{-1})	0.75 ± 0.12	0.27 ± 0.07
K_M (nM)	60 ± 17	7.7 ± 1.8
k_{cat}/K_M ($s^{-1}M^{-1}$)	1.2 ± 0.2 x 10^7	3.8 ± 1.5 x 10^7
	**ATP**	
	WT	E346A/E592A
k_{cat} (s^{-1})	0.83 ± 0.01	0.35 ± 0.03
K_M (μM)	9.2 ± 1.8	23 ± 3
k_{cat}/K_M ($s^{-1}M^{-1}$)	9.4 ± 1.7 x 10^4	1.6 ± 0.3 x 10^4

Supplementary Table 2. Steady-state kinetic parameters for WT and mutant LIG1 at 20 mM Mg^{2+}. These data are from the experiments in Figure S2.

	WT	EE/AA
k_{cat} (s^{-1})	0.72 ± 0.03	0.25 ± 0.06
K_{Mg} (mM)	0.78 ± 0.10	0.78 ± 0.05
$k_{transfer}$ (s^{-1})	3.0 ± 0.6	5.3 ± 1.2
$K_{Mg,transfer}$ (mM)	0.10 ± 0.03	≤ 0.1
k_{seal} (s^{-1})	16 ± 4	27 ± 9
$K_{Mg,seal}$ (mM)	4.9 ± 2.1	9 ± 4

Supplementary Table 3. Kinetic parameters for varying the concentration of Mg^{2+}. These data for WT and mutant LIG1 are from the fits in Figure S3 for the 28mer-C:G substrate.

	28mer-C:G		28mer-8oxoG:C		28mer-8oxoGA	
	WT	E346A/E592A	WT	E346A/E592A	WT	E346A/E592A
k_{cat} (s^{-1})	0.42 ± 0.04	0.20 ± 0.03	7.2 ± 0.8 x 10^{-3}	3.7 ± 0.8 x 10^{-3}	0.11 ± 0.01	0.072 ± 0.010
K_M (nM)	79 ± 14	3.3 ± 0.7	132 ± 21	2.6 ± 0.7	78 ± 14	5.1 ± 3.0
k_{cat}/K_M ($s^{-1}M^{-1}$)	5.5 ± 0.9 x 10^6	6.0 ± 0.8 x 10^7	5.3 ± 0.4 x 10^4	1.6 ± 0.4 x 10^6	1.5 ± 0.2 x 10^6	1.9 ± 1 x 10^7
$k_{transfer}$ (s^{-1})	3.6 ± 0.9	7.5 ± 2.0	0.011 ± 0.002	0.010 ± 0.002	0.77 ± 0.07	1.0 ± 0.1
k_{seal} (s^{-1})	3.1 ± 0.3	3.7 ± 0.7	0.0085 ± 0.0022	0.0058 ± 0.0016	0.071 ± 0.008	0.18 ± 0.01

Supplementary Table 4. Kinetic parameters for ligation of DNA substrates at 1 mM Mg^{2+}_{free}. The steady-state kinetic data is from Figure S4 and the single-turnover kinetic data is from Figure 3D.
